# Fluorescence Lifetime Spectroscopy Reveals Distinct Allosteric Mechanisms of SERCA Inhibitors

**DOI:** 10.1007/s00232-026-00380-9

**Published:** 2026-05-20

**Authors:** Jaroslava Šeflová, Carlos Cruz-Cortés, Seth L. Robia, L. Michel Espinoza-Fonseca

**Affiliations:** 1 Department of Cell and Molecular Physiology, Loyola University Chicago, Maywood, IL 60153 USA; 2 Department of Internal Medicine, Division of Cardiovascular Medicine, University of Michigan, Ann Arbor, MI 48109 USA; 3 Center for Arrhythmia Research, University of Michigan, Ann Arbor, MI 48109 USA

**Keywords:** SERCA, Fluorescence lifetime spectroscopy, Allosteric modulation, Inhibition

## Abstract

**Abstract:**

We recently developed a time-correlated single photon counting (TCSPC) spectroscopy approach to investigate the activation mechanisms of the calcium pump SERCA (sarcoplasmic reticulum Ca^2+^-ATPase). Here, we apply this approach to characterize the effects of two chemically distinct SERCA inhibitors, thapsigargin (TG) and cyclopiazonic acid (CPA), and to determine how they differentially modulate SERCA conformational dynamics. Although TG and CPA appear to stabilize similar SERCA states in structural studies, TCSPC reveals fundamentally distinct mechanisms of inhibition. TG stabilizes an inactive conformation that prevents Ca^2+^- and ATP-dependent transitions, effectively trapping SERCA in a nonproductive ‘dead-end’ state. In contrast, CPA attenuates, rather than abolishes, Ca^2+^- and nucleotide-dependent structural transitions, producing graded, concentration-dependent effects that reduce the population of the closed, activation-associated state. Notably, CPA decreases apparent Ca^2+^ affinity only in the presence of a non-hydrolyzable ATP analog, consistent with an ATP-dependent, allosteric mechanism that redistributes conformational populations rather than directly occluding Ca^2+^ binding sites. These findings demonstrate that TCSPC resolves mechanistic differences between inhibitors that appear structurally similar and provide a framework for understanding how distinct allosteric ligands modulate SERCA function.

**Graphical Abstract:**

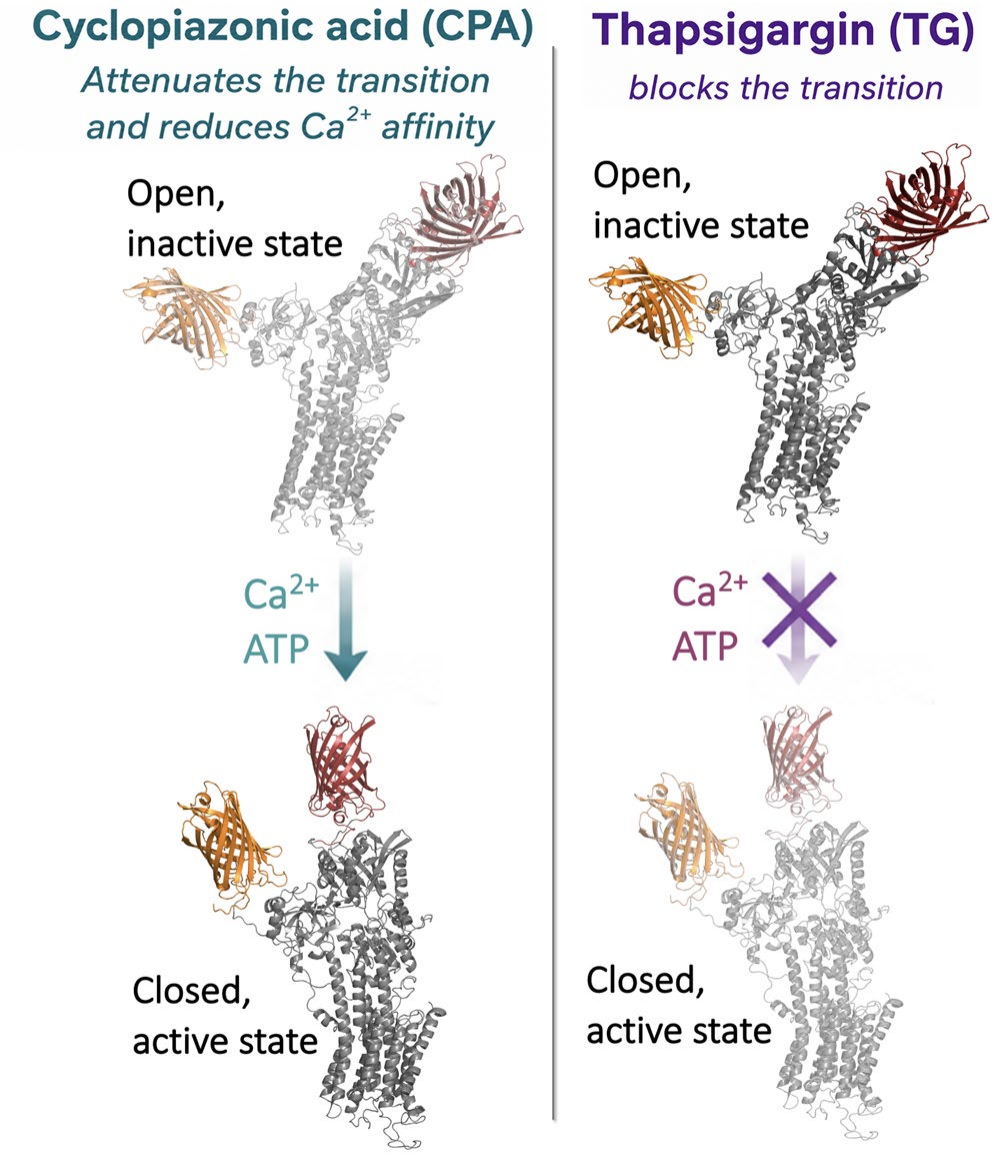

## Introduction

**Fig. 1:**
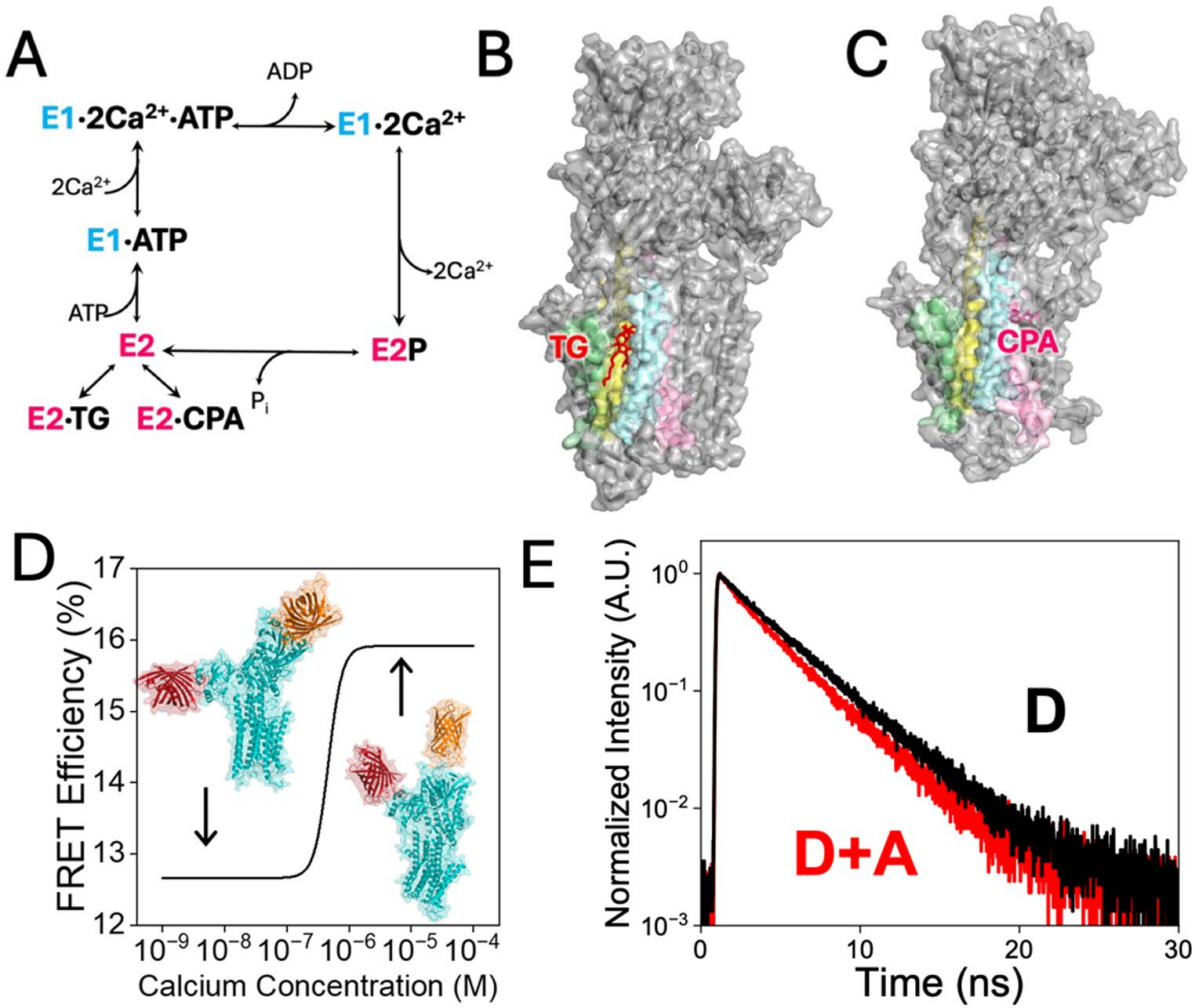
Intermediate states and crystal structures of SERCA stabilized by TG and CPA. (**A**) Schematic representation of the Post–Albers pumping cycle of SERCA, highlighting intermediate states preferentially populated by thapsigargin (TG) and cyclopiazonic acid (CPA) based on crystallographic evidence. Crystal structures of SERCA bound to (**B**) TG (PDB: 2AGV) and (**C**) CPA (PDB: 2EAU) illustrate the distinct locations of their binding sites. In all panels, SERCA is shown as a gray surface with selected transmembrane helices highlighted: TM3 (cyan), TM4 (magenta), TM5 (yellow), and TM7 (green). The inhibitors are depicted as stick representations, colored red (TG) and magenta (CPA). (**D**) Representative FRET changes measured using the two-color SERCA construct. At low Ca^2+^ concentrations, the dynamic equilibrium among SERCA conformational states is shifted toward an open conformation, corresponding to low FRET efficiency. At high Ca^2+^ concentrations, the equilibrium shifts toward a closed conformation, resulting in higher FRET efficiency. Two representative SERCA structures (PDB: 1SU4 and 2ZBD) are shown in teal, with the fluorescent proteins mCyRFP1 (orange) and mMaroon1 (dark red) indicated. (E) Representative fluorescence decay curves for mCyRFP1 alone (black, donor only, D) and the two-color SERCA construct (red, donor + acceptor, D + A)

The sarcoplasmic reticulum Ca^2+^-ATPase (SERCA) is an essential component of the sarcoplasmic reticulum (SR) membrane in muscle cells (Periasamy and Huke 2001). SERCA-mediated SR Ca^2+^ uptake maintains a 10,000-fold calcium gradient across the SR membrane and plays a key role in muscle relaxation (Toyoshima 2009). SERCA counter-transports two Ca^2+^ ions and 2–3 protons using energy derived from the hydrolysis of ATP (Yu et al. 1993; Zafar et al. 2008). The catalytic cycle of SERCA begins with the formation of the high-Ca^2+^ affinity E1 state that binds two Ca^2+^ ions and one molecule of ATP (Aguayo-Ortiz and Espinoza-Fonseca 2020). This Ca^2+^/ATP-bound E1 state facilitates ATP hydrolysis and SERCA autophosphorylation, yielding an intermediate E1 ~ P-ADP-2Ca^2+^ state (Toyoshima and Mizutani 2004). This step in the catalytic cycle induces a structural transition toward a phosphorylated, low-Ca^2+^ affinity E2 state E2P-2Ca^2+^ that facilitates the release of Ca^2+^ ions into the SR lumen. Upon Ca^2+^ release, two luminal protons diffuse into the transport sites to stabilize the phosphorylated E2-P state (Obara et al. 2005). This step is followed by SERCA dephosphorylation required to populate the E2 state of the pump (Olesen et al. 2004). Spontaneous proton release to the cytosol then facilitates the E2-to-E1 transition, thus populating the E1 state for the next Ca^2+^-pumping cycle (Aguayo-Ortiz and Espinoza-Fonseca 2020). A diagram of the catalytic cycle of SERCA is shown in Fig. 1A.

Small molecules that allosterically inhibit SERCA have been found predominantly in two distinct sites of the protein: the thapsigargin (TG)-binding site (Fig. 1B), and the doorstop inhibitor binding site that is located near the cytosolic Ca^2+^ entry gate (Fig. 1C) (Aguayo-Ortiz and Espinoza-Fonseca 2020). The TG-binding site is found in a transmembrane pocket formed by transmembrane helices M3, M4, M5, and M7 of the pump (Winther et al. 2010). Biochemical studies have shown that binding of TG to this site stabilizes a dead-end, catalytically inactive E2-like state of SERCA (Fig. 1A) (Sagara et al. 1992). More recently, we showed that TG stabilizes a rigid open structure of SERCA’s headpiece that abolishes pumps’ headpiece closing in response to increasing Ca^2+^ concentration, effectively impeding the formation of SERCA structures in the E1 state that are primed for phosphorylation (Seflova et al. 2024). The doorstop inhibitor site, which is formed by the SERCA’s transmembrane helices M1, M2, M3, and M4, binds cyclopiazonic acid (CPA), a well-studied allosteric inhibitor of the pump (Moncoq et al. 2007). The binding of CPA to the doorstop inhibitor site (Fig. 1C) also stabilizes an E2-like state of the pump (Fig. 1A) (Laursen et al. 2009; Moncoq et al. 2007). Specifically, crystallography and biochemical studies have proposed that CPA blocks Ca^2+^ access to the transport sites and affects the nucleotide-mediated activation of SERCA (Moncoq et al. 2007). Therefore, TG and CPA occupy two distinct binding sites and stabilize E2-like SERCA states, but the mechanism of the inhibition for each compound is dissimilar.

X-ray crystallography studies have shown that both TG and CPA stabilize similar SERCA structural states (Aguayo-Ortiz and Espinoza-Fonseca 2020), yet their differential effects on conformational populations and Ca^2+^ affinity remain poorly understood. Because crystallographic approaches provide static snapshots, they do not resolve how distinct inhibitors reshape the dynamic conformational equilibria of SERCA under varying ligand conditions. To address this limitation, we recently developed a time-correlated single photon counting (TCSPC) approach to quantify Förster resonance energy transfer (FRET) between two fluorescent protein tags fused to the SERCA cytoplasmic headpiece (Seflova et al. 2024) (Fig. 1D). In contrast to static structural methods, TCSPC directly measures ligand-dependent shifts in conformational populations, enabling discrimination between allosteric mechanisms that may appear structurally similar. FRET efficiency reports on the donor–acceptor distance and thus serves as a quantitative readout of the overall conformation of the transporter (Fig. 1D). Specifically, TCSPC determines FRET by measuring the fluorescence lifetime of the donor fluorophore, which decreases in the presence of an acceptor (Fig. 1E). Structural transitions that bring the donor and acceptor into closer proximity increase FRET and further shorten the donor lifetime. We have applied this approach to characterize structural transitions between enzymatic states (Raguimova et al. 2018) and to define the mechanisms of allosteric modulation by small molecules (Cleary et al. 2024; Seflova et al. 2024), including Ca^2+^- and ATP-dependent activation of SERCA through redistribution of conformational populations. In this study, we use TCSPC to interrogate how CPA inhibits SERCA in response to Ca^2+^ and ATP, in direct comparison with TG. Despite their similar crystallographic endpoints, we find that these inhibitors act through fundamentally distinct allosteric mechanisms: CPA disrupts the ATP-mediated activation pathway, whereas TG stabilizes an inactive conformation that effectively blocks Ca^2+^- and ATP-dependent transitions. These findings reveal distinct allosteric mechanisms of inhibition, providing new insights into the modulation of SERCA by chemically diverse small-molecule effectors.

## Materials and Methods

*Chemicals.* All chemicals used in this study were purchased at reagent quality (purity > 95% by HPLC): Thapsigargin (Sigma, St. Louis, MO); cyclopiazonic acid (Sigma, St. Louis, MO); and AMP-PCP (β,γ-Methyleneadenosine-5’-triphosphate; Sigma, St. Louis, MO).

*Construct generation and HEK-293T cell culture.* The generation of 2-color cardiac SERCA constructs was described previously by (Cleary et al. 2024; Raguimova et al. 2018; Seflova et al. 2024). Briefly, 2-color canine SERCA2a was produced by introducing mMaroon1 to the N-terminus of SERCA pump using the BglI restriction enzyme. This mMaroon1 construct yielded mMaroon1 fused to N-terminus of SERCA pump. Subsequently, mCyRFP1 was introduced into the N-domain of SERCA pump at position 509 using HindIII restriction enzyme. The final constructs were verified by sequencing (ACGT, Wheeling, IL). The human type 5 (dE1/E3) adenovirus was subcloned into the adenoviral D2-MCS vector with CMV promoter by Vector Biolabs (Malvern, PA) using NheI/KpnI insert from the original 2-color SERCA construct described above. The adenovirus was stored in DMEM supplemented with 2% BSA and 2.5% glycerol, and the PFU titer was determined as 2.2 × 10^10^ PFU/ml. HEK 293T cells (CRL-3216, ATCC) were seeded into a 150 mm cell culture dish 72 h before viral infection and cultured in DMEM with 4.5 g/L glucose, L-glutamine and sodium pyruvate (Corning, USA) supplemented with 10% heat inactivated FBS (Corning, USA) in 5% CO_2_ incubator at 37 °C. When cells reached 90% confluence, the cell culture media was replaced with fresh media. Confluent cells were infected with an adenovirus encoding 2-color SERCA. The multiplicity of infection (MOI) was 35, determined *via* a small-scale infection screen. Infected cells were harvested 48 h post-infection.

*Microsomal membranes preparation from HEK-293T cells infected with adenovirus.* Microsomal membranes were prepared following a protocol published previously (Raguimova et al. 2018, 2020). Specifically, each 150 mm cell culture dish was scraped into 12 ml of homogenization buffer containing 0.5 mM MgCl_2_, 10 mM Tris-HCl (pH 7.5), and supplemented with UltraCruz Protease Inhibitor Cocktail Tablet without EDTA (Santa Cruz Biotechnology, Dallas, TX). Cells were pelleted by centrifugation at 1,000 *g* for 10 min at 4 °C and subsequently resuspended in 5 ml of fresh homogenization buffer. The cell suspension was homogenized with 20 strokes of Potter-Elvehjem glass homogenizer, and 5 ml of sucrose buffer (100 mM MOPS, 500 mM sucrose; pH 7.0). The homogenate was passed 10 times through a 27-gauge needle. Subsequently, the homogenate was centrifuged at 1,000 *g* for 10 min at 4 °C to remove unbroken cells, mitochondria, and cellular debris. The supernatant was subjected to ultracentrifugation at 126,000 *g* for 30 min at 4 °C, and the pellet was dissolved in a 1:1 mixture of homogenization and sucrose buffers. The total protein concentration was determined using BCA assay (Pierce BCA Protein assay, ThermoFisher Scientific, Rockford, IL).

*Time-correlated single-photon counting spectroscopy of SERCA.* For TCSPC measurements, microsomes were thawed on ice, mixed with the DMSO vehicle (0.01%) or the inhibitors TG or CPA (> 98% purity by HPLC; Sigma-Aldrich), and incubated for 30 min at room temperature. The microsomes were then mixed with a buffer (120 mM potassium aspartate, 15 mM KCl, 5 mM KH_2_PO_4_, 0.75 mM MgCl_2_, 2% dextran, 20 mM HEPES, 2 mM EGTA, and 1.7 mM CaCl_2_; pH 7.2) with or without 500 µM AMP-PCP. TCSPC was performed immediately after mixing. The free Ca^2+^ concentrations (1 nM to 100 µM) of these solutions were calculated using MaxChelator (Schoenmakers et al. 1992). The solutions containing microsomes were pipetted onto the surface of a chambered coverslip (Cellvis, Mountain View, CA) for time-correlated single photon counting (TCSPC) measurements, as previously described (Seflova et al. 2024). Briefly, fluorescence excitation was accomplished with a 20 MHz supercontinuum pulsed laser (SuperK Evo, NKT Photonics, Denmark). The focused laser was positioned inside the microsome drop, yielding a count rate of 10,000 photons/s. Under those conditions, we observed less than 10% photobleaching during the 60 s of acquisition time. Fluorescence was detected through a 1.2 NA water-immersion objective using a PMA hybrid detector (PicoQuant, Berlin, Germany) connected to a single photon-counting module (HydraHarp 300, PicoQuant, Berlin, Germany) with a time channel width of 16 ps. The donor (mCyRFP1) signal was acquired using an excitation bandpass filter 482/18 nm in the excitation laser pathway. This arrangement provided 4 orders of magnitude difference between signal and noise (Seflova et al. 2024). Using microsomes prepared from untransfected cells or cells expressing the unlabeled (non-fluorescent) pump we detected in total ~ 10,000 photons of background autofluorescence, with an average lifetime of 2.4 ns. Thus, in cells expressing 2-color SERCA, we estimate that the background autofluorescence accounts for ~ 2% of total photons.

The time delay between the excitation laser pulse and the detection of the resulting photon was measured to build a histogram of photon arrival times that revealed the shape of the donor fluorescence decay on a nanosecond timescale. Several million photon arrival times were quantified during the 60 s acquisition time to reconstruct each fluorescence decay. The measured decay curve was globally fitted using single or multi-exponential models using SymPhoTime 64 software (Picoquant, Berlin, Germany). The distribution of fit residuals and χ^2^ of the fit were used to evaluate the appropriate number of parameters to include in the fit. The fluorescence decay of singly-labeled mCyRFP1-SERCA was well-described by a single exponential fit for measurements made in intact HEK293T cells as well as in microsomal fractions prepared from those cells. In some cases, the decay fit slightly better with a two-exponential decay model, but the contribution of the second component was less than 10% of the total amplitude of the decay. 2-color SERCA was characterized by a shorter, multi-exponential decay (Fig. 1E) consistent with intramolecular FRET. The decays were analyzed with a two-component fit representing a simplified model of two conformations, an “open” non-FRET conformation with a fluorescence lifetime of 3.5 ns, and a “closed” FRET conformation (Fig. 1D) with a fluorescence lifetime of 1.7 ns. The addition of the third component did not yield a substantial improvement in the residual distribution or χ^2^. The relative contribution of the two components to the overall average amplitude-weighted lifetime was quantified to reveal changes in dynamic equilibrium between open and closed states (Raguimova et al. 2020; Seflova et al. 2024).

*Data analysis and statistics*. The apparent Ca^2+^ affinity was determined by nonlinear regression of the FRET efficiency–Ca^2+^ concentration relationship using a four-parameter Hill equation. The lower and upper FRET plateaus, Hill coefficient, and half-maximal Ca^2+^ concentration were allowed to vary during fitting. The Ca^2+^ concentration corresponding to 50% of the total FRET change was defined as K_Ca_. All results are presented as mean ± standard error of the mean (SEM). Statistical significance was evaluated using two-way analysis of variance (ANOVA) followed by Dunnett’s post hoc test for multiple comparisons against the control. We used 95% confidence intervals around the differences between the groups for the post-hoc test. Two-sided *p* values were used, and α-level < 0.05 was considered significant.

## Results

### Thapsigargin Inhibits the Response of SERCA to both Ca^2+^ and the Non-hydrolyzable ATP analog AMP-PCP

**Fig. 2:**
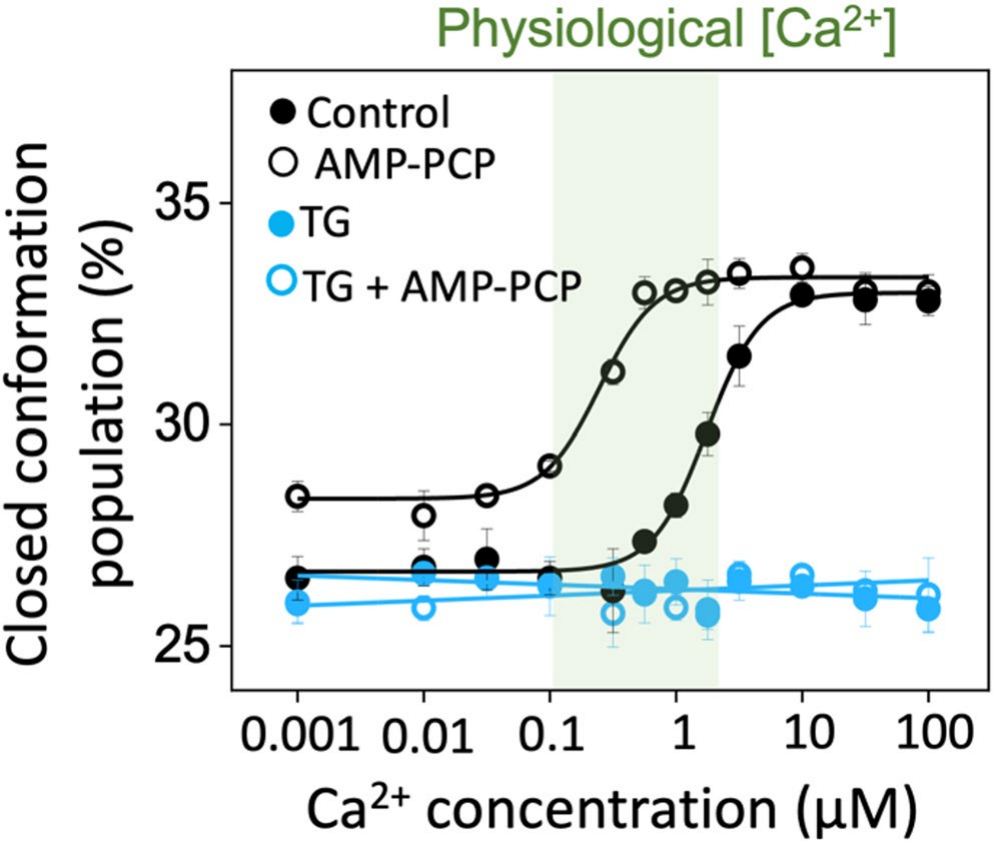
Structural changes of SERCA in response to thapsigargin. 12-point Ca^2+^ concentration-response curves in response to TG (10 µM) were obtained in the absence (closed symbols) and presence (open symbols) of AMP-PCP (500 µM). The structural change in response to ligands is shown as the % of the closed population of the headpiece. The green shaded area indicates the physiological range of Ca^2+^ concentrations. Data are reported as average ± SEM of four biological replicates (*N* = 4 biological replicates)

We previously reported the effects of TG in microsomes from HEK-293T cells transfected with fluorescently labeled SERCA; here, we used 2-color SERCA expressed via adenoviral infection to increase protein yield and evaluated TG effects in microsomal membranes from infected cells. In the absence of TG, TCSPC measurements show a Ca^2+^-dependent increase in the closed-state population of SERCA in a Ca^2+^ concentration-dependent manner, with the population of the closed state of SERCA shifting from ~ 26.5% at nanomolar Ca^2+^ concentrations to ~ 33% at saturating micromolar Ca^2+^ concentrations (Fig. 2, black). This Ca^2+^-dependent response is similar to that observed in SERCA-transfected cells (Seflova et al. 2024). In agreement with our previous study using transfected HEK-293T cells, we found that the addition of 10 µM TG completely blunts the Ca^2+^-dependent shift of SERCA populations (Fig. 2, blue), and this effect is not reversed even at supra-physiological Ca^2+^ concentrations, e.g., 100 µM Ca^2+^ (Fig. 2). In the absence of TG, addition of AMP-PCP substantially increases the fraction of SERCA in the closed conformation at nanomolar Ca^2+^ concentrations (Fig. 2, black open symbols). Conversely, 10 µM TG eliminates the Ca^2+^-dependent FRET response, consistent with suppression of conformational transitions (Fig. 2, blue open symbols). These observations agree with our recent findings and correlate with studies showing that TG inhibits calcium loading (Thastrup et al. 1990) and the formation of catalytically competent SERCA structures (Raguimova et al. 2018). The qualitative agreement between these results and our previous study indicates that the response of SERCA to Ca^2+^, AMP-PCP, and TG is similar between microsomes from transfected and infected HEK-293T cells. Importantly, consistent with our prior work (Seflova et al. 2024), TG produces a near-complete suppression of the Ca^2+^-dependent transition even at low micromolar concentrations, yielding a largely flat response and precluding partial conformational shifts.

## CPA Induces Nucleotide-independent Allosteric Effects on SERCA

**Fig. 3:**
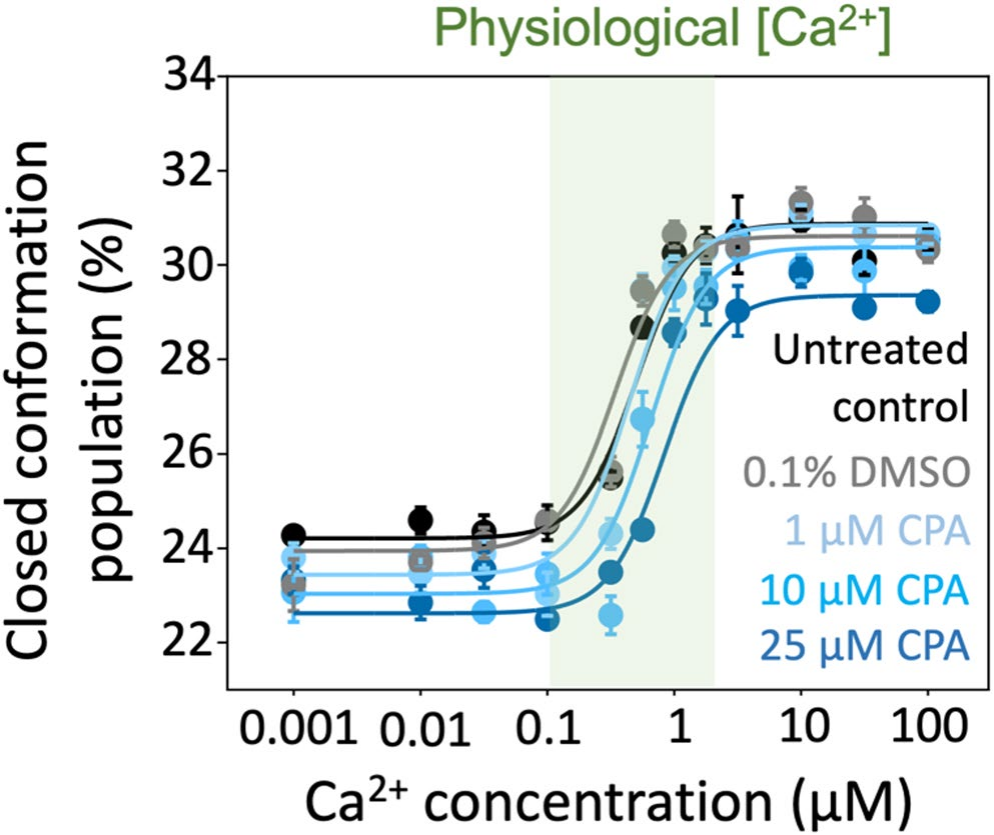
Ca^2+^ concentration-dependent structural changes of SERCA in response to CPA and the absence of AMP-PCP. Twelve-point Ca^2+^ concentration–response curves were measured in the presence of CPA at three inhibitor concentrations. Untreated SERCA and DMSO served as controls, and to exclude the potential effects of the DMSO-containing vehicle on the response to Ca^2+^. The green shaded area indicates the physiological range of Ca^2+^ concentrations. Data are reported as the percentage of the closed headpiece population are shown as mean ± SEM (*N* = 4 biological replicates)

We evaluated the effects of CPA on SERCA in the absence of nucleotide to clarify allosteric effects that occur independently of ATP. Initial experiments indicated that the effects observed in the presence of CPA were more subtle than those induced by TG. Therefore, to rule out potential vehicle effects on SERCA structural dynamics, we first tested the [Ca^2+^]-dependent responses of SERCA in the absence and presence of DMSO. We found that SERCA’s response to increasing Ca^2+^ concentrations is virtually identical in the presence or absence of DMSO (Fig. 3, black and gray traces), indicating that the vehicle does not affect the signal. Next, we tested concentrations 1, 10, and 25 µM of CPA in a [Ca^2+^]-dependent manner. We found that at CPA concentrations of 1, 10, and 25 µM, SERCA’s response to increasing Ca^2+^ concentrations follows a sigmoidal relationship, with a shift in SERCA populations toward the closed state similar to that of untreated controls (Fig. 3). However, CPA at concentrations of 10 and 25 µM partially blunts the Ca^2+^- dependent increase in FRET amplitude, consistent with a decreased population of the closed conformation (Fig. 3). Importantly, this effect correlates with increasing CPA concentrations, indicating that the inhibitory impact on SERCA’s conformational shift is concentration dependent.

**Fig. 4:**
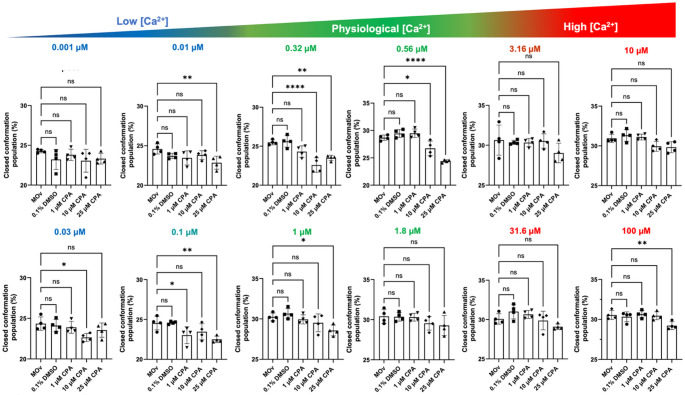
Effects of CPA on the structural populations of SERCA in the absence of AMP-PCP. We analyzed the effects of three different CPA concentrations on SERCA’s response to Ca^2+^. Untreated SERCA (MOv) and DMSO served as controls, and to exclude the potential effects of the DMSO-containing vehicle on the response to Ca^2+^. The physiological window is shown in green, and low and high Ca^2+^ concentrations falling outside this window are shown in blue and red, respectively. Data are shown as mean ± SEM of four biological replicates (*N* = 4). We used a two-way ANOVA followed by Dunnett’s post-hoc test to compare treatments against the untreated SERCA (MOv). *p < 0.05; **p < 0.01; ***p < 0.001; **** *p* < 0.0001; ns, not significant

We next tested the hypothesis that in the absence of ATP, CPA alone has a significant Ca^2+^-mediated allosteric effect on SERCA. We analyzed the changes in SERCA populations in response to CPA using a two-way ANOVA and the post-hoc Dunnett’s test to compare the changes in SERCA populations at twelve increasing Ca^2+^ concentrations. The statistical analysis showed that, compared to the untreated control, DMSO or 1 µM CPA do not have a significant effect on the headpiece population at most Ca^2+^ concentrations (Fig. 4). The only significant effect was observed for 1 µM CPA at [Ca^2+^] = 0.1 µM, which represents the lower limit of the physiological range of Ca^2+^ concentrations at which SERCA is operational in cardiac cells (Cheung et al. 1989; Fearnley et al. 2011; Kirschenlohr et al. 2000). Conversely, 10 µM CPA significantly reduces the FRET-derived shift in headpiece populations toward the closed conformation across most Ca^2+^ concentrations between 0.03 and 0.56 µM, with the most significant effect at [Ca^2+^] = 0.32 µM (Fig. 4). However, 10 µM CPA had no effect on the Ca^2+^-mediated structural shifts of SERCA at saturating Ca^2+^ concentrations (Fig. 4), indicating that inhibition of these transitions occurs only at or below physiological Ca^2+^ levels. CPA at a concentration of 25 µM had a similar effect on the SERCA populations, significantly inhibiting the open-to-closed transition across most Ca^2+^ concentrations between 0.01 and 1 µM (Fig. 4). The most significant effect of 25 µM CPA was observed at a Ca^2+^ concentration of 0.56 µM (Fig. 4). Interestingly, we found that CPA exerts a significant effect on SERCA populations at a Ca^2+^ concentration of 100 µM, indicating that inhibition of the Ca^2+^-induced structural transitions of SERCA can occur even at saturating calcium levels. Importantly, we found that the effects of CPA on the populations of SERCA occur primarily at Ca^2+^ concentrations within the physiological window at which SERCA operates (Cheung et al. 1989; Fearnley et al. 2011; Kirschenlohr et al. 2000) (Figs. 3 and 4). Collectively, these findings indicate that CPA alone modulates SERCA’s structural populations independently of nucleotide.

## Modulation of SERCA by CPA in the Presence of the ATP analog AMP-PCP

**Fig. 5:**
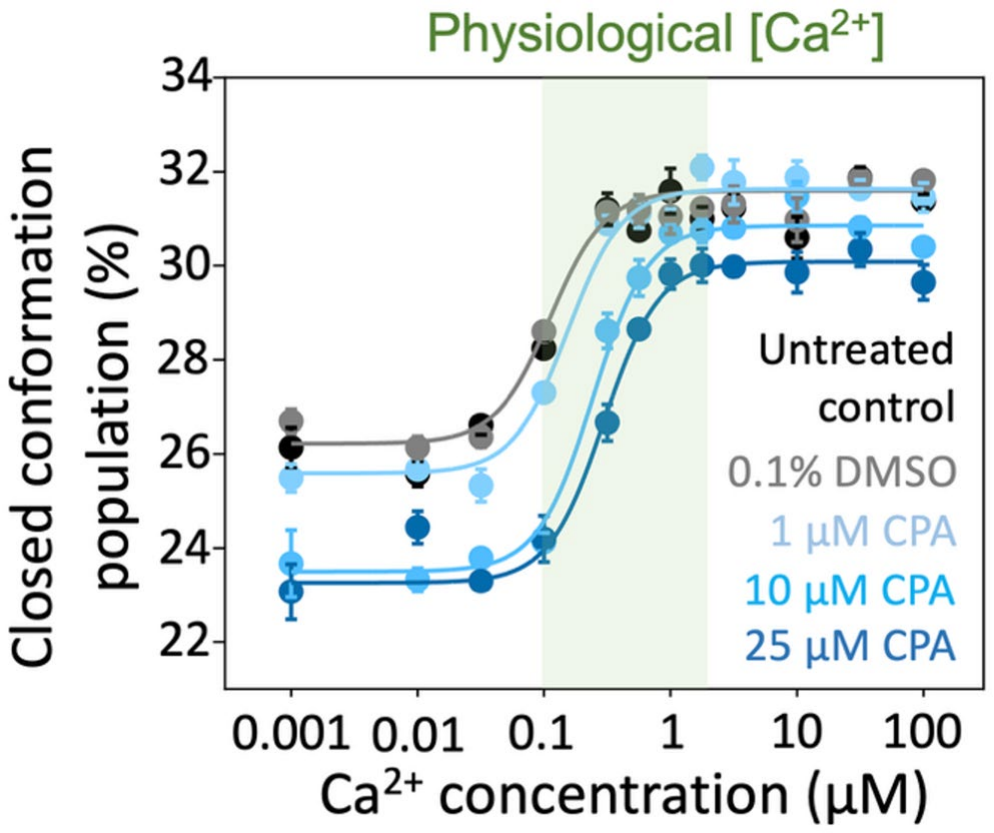
Ca^2+^ concentration-dependent structural changes of SERCA in response to CPA in the presence of the ATP analog AMP-PCP. Twelve-point Ca^2+^ concentration–response curves were measured in the presence of AMP-PCP (500 µM) and CPA at three concentrations. Untreated SERCA and DMSO served as controls, and to exclude the potential effects of the DMSO-containing vehicle on the response to Ca^2+^. The green shaded area indicates the physiological range of Ca^2+^ concentrations. Data are reported as the percentage of the closed headpiece population are shown as mean ± SEM (*N* = 4 biological replicates)

We next evaluated the combined effects of CPA (1, 10, and 25 µM) and ATP on the structural dynamics of the SERCA headpiece. To prevent ATP hydrolysis from confounding the results, these experiments were conducted using the non-hydrolyzable analog AMP-PCP. Similar to the nucleotide-free experiments, we first tested the response of SERCA to AMP-PCP in the absence and presence of DMSO. We found that SERCA’s response to AMP-PCP and increasing Ca^2+^ concentrations is virtually identical in the presence or absence of DMSO (Fig. 5, black and gray traces), indicating that the vehicle does not affect the signal. Importantly, AMP-PCP increases the fraction of the closed state of SERCA at physiologically relevant Ca^2+^ concentrations in cardiac cells, in agreement with previous studies by our group (Cleary et al. 2024; Seflova et al. 2024). We found that in the presence of AMP-PCP, there is a sigmoid relationship between Ca^2+^ concentrations at all three concentrations of CPA tested (Fig. 5). Compared with the untreated control, 1 µM CPA decreases the population of SERCA in the closed conformation across most Ca^2+^ concentrations tested, without inducing a rightward shift of the curve (Fig. 5). Conversely, CPA at concentrations of 10 and 25 µM reduces the FRET-derived closed-state population of SERCA across all Ca^2+^ concentrations and a rightward shift of the curve (Fig. 5). As observed in the absence of AMP-PCP, this effect correlates with increasing CPA concentrations, indicating that the inhibitory impact on SERCA’s conformational shift is concentration-dependent.

**Fig. 6:**
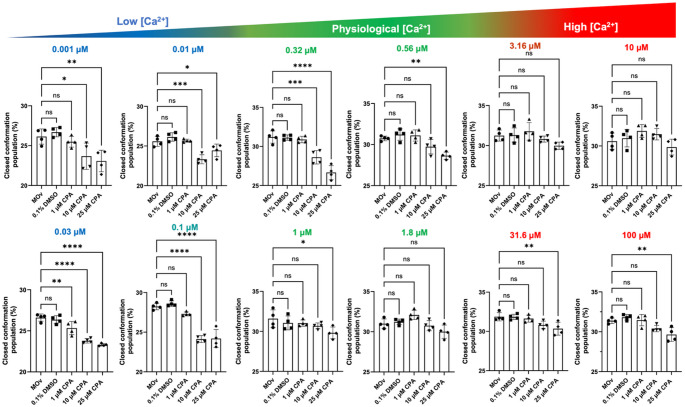
Effects of CPA on the structural populations of SERCA in the presence of AMP-PCP. We analyzed the effects of three different CPA concentrations on SERCA’s response to Ca^2+^ and AMP-PCP (500 µM). Untreated SERCA (MOv) and DMSO served as controls, and to exclude the potential effects of the DMSO-containing vehicle on the response to Ca^2+^. The physiological window is shown in green, and low and high Ca^2+^ concentrations falling outside this window are shown in blue and red, respectively. Data are shown as mean ± SEM of four biological replicates (*N* = 4). We used a two-way ANOVA followed by Dunnett’s post-hoc test to compare treatments against the untreated control (MOv). *p < 0.05; **p < 0.01; ***p < 0.001; **** *p* < 0.0001; ns, not significant

To assess how CPA modulates SERCA headpiece dynamics in the presence of nucleotide, we examined Ca^2+^-dependent changes in the closed conformation population in the presence of the non-hydrolyzable ATP analog AMP-PCP. Under control conditions (untreated microsomes, MOv, and DMSO), SERCA exhibited the expected Ca^2+^-dependent increase in the closed conformation population across the full Ca^2+^ range tested. At 1 µM CPA, the fraction of SERCA in the closed conformation was not significantly reduced (except at [Ca^2+^] = 0.03 µM) (Fig. 6). In contrast, CPA at 10 µM significantly decreases the closed conformation population across most Ca^2+^ concentrations between 0.001 and 0.32 µM (Fig. 6). The effects of 10 µM CPA were most pronounced at low and physiological Ca^2+^ concentrations (≤ 0.32 µM), whereas the inhibitory effects of CPA were abolished by Ca^2+^ concentrations ≥ 0.56 µM (Fig. 6). CPA at a concentration of 25 µM also significantly inhibits the open-to-closed transition across most Ca^2+^ concentrations between 0.001 and 1 µM (Fig. 6). The most significant effect of 25 µM CPA was observed at a Ca^2+^ concentrations of 0.03, 0.1, and 0.32 µM (Fig. 6). Consistent with the nucleotide-free experiments, CPA significantly inhibits the open-to-closed transition of SERCA at Ca^2+^ concentrations of 31.6 and 100 µM, indicating that CPA-mediated suppression of Ca^2+^-induced structural transitions persists even in the presence of AMP-PCP and saturating Ca^2+^ conditions. Together, these data demonstrate that CPA antagonizes Ca^2+^-dependent headpiece closure even in the presence of nucleotide, revealing a nucleotide-independent allosteric mechanism that becomes increasingly dominant at higher CPA concentrations.

## CPA alters ATP-dependent Ca^2+^ affinity of SERCA

**Fig. 7:**
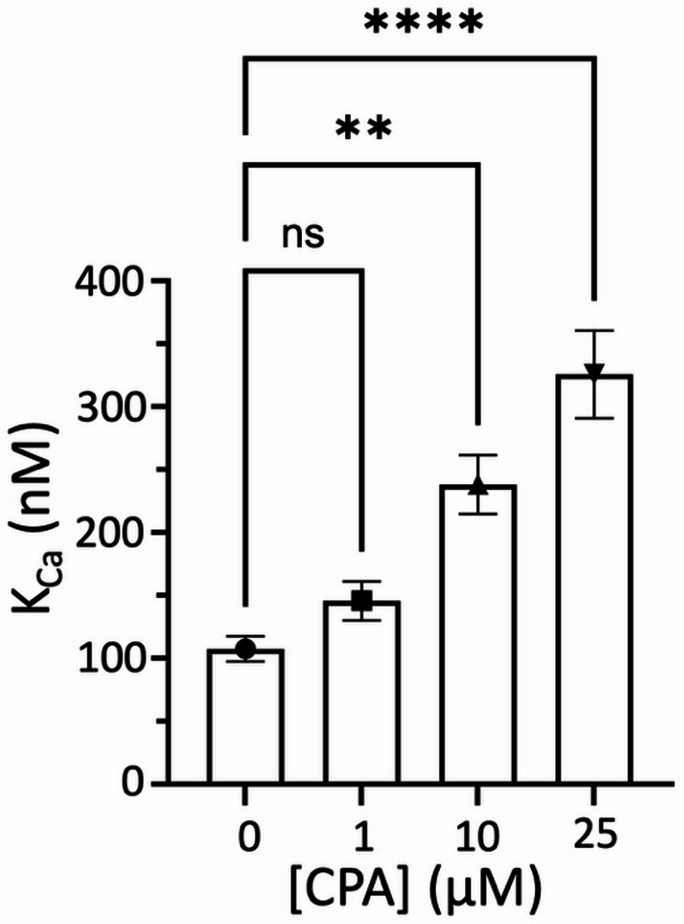
CPA alters ATP-dependent Ca^2+^ affinity of SERCA. Changes in equilibrium Ca^2+^ affinity (K_Ca_) induced by increasing concentrations of CPA in the presence of the non-hydrolyzable ATP analog AMP-PCP (500 µM). Bars represent mean ± SEM (*N* = 4 biological replicates). Statistical significance was assessed using a two-way ANOVA followed by Dunnett’s post-hoc test to compare treatments against the control (e.g., in the absence of CPA). **p < 0.01; **** *p* < 0.0001; ns, not significant

In the absence of AMP-PCP, no systematic shift in the Ca^2+^-dependent curves was observed across conditions (Fig. 3). Consistent with this, the apparent Ca²⁺ affinity was not significantly different between groups, with midpoint values clustering around ~ 0.6–0.8 µM. These results indicate that increasing CPA does not measurably alter Ca^2+^ sensitivity under nucleotide-free conditions. In contrast, in the presence of the non-hydrolyzable ATP analog AMP-PCP, CPA induced a rightward shift in the Ca^2+^ concentration–response curves, indicative of reduced apparent Ca^2+^ affinity (Fig. 5). To quantify this effect, we extracted Ca^2+^ affinity constants (K_Ca_) from TCSPC data. Under control conditions (MOv and DMSO), SERCA exhibited a K_Ca_ of approximately 100 nM. Addition of 1 µM CPA produced a modest, non-significant increase in K_Ca_ (Fig. 7), indicating that low CPA concentrations do not measurably perturb Ca^2+^ binding in the nucleotide-bound state. In contrast, higher CPA concentrations significantly reduced apparent Ca^2+^ affinity. At 10 µM CPA, K_Ca_ increased to ~ 240 nM (~ 2.4-fold decrease in affinity), and at 25 µM to ~ 320 nM (~ 3.2-fold decrease) (Fig. 7). These effects were statistically significant and dose-dependent, consistent with progressive destabilization of high-affinity Ca²⁺ binding with increasing CPA concentration. These data favor an ATP-dependent allosteric mechanism rather than direct occlusion of the Ca^2+^ binding sites by CPA. Together, these results indicate that CPA attenuates, rather than abolishes, Ca^2+^- and nucleotide-dependent structural transitions of SERCA.

## Discussion

In this study, we used TCSPC spectroscopy (Cleary et al. 2024; Seflova et al. 2024) to directly resolve how chemically distinct allosteric inhibitors TG and CPA reshape the conformational landscape of SERCA under defined Ca^2+^- and nucleotide-binding conditions. This approach enabled us to interrogate inhibitor-specific effects on structural populations, revealing fundamental mechanistic differences between chemically distinct SERCA inhibitors.

TG is a sesquiterpene lactone that inhibits SERCA by binding to a large pocket in the transmembrane domain of the pump (Aguayo-Ortiz and Espinoza-Fonseca 2020). We found that TG produced a complete inhibition of Ca^2+^-dependent structural transitions, consistent with its well-established role as a high-affinity, irreversible inhibitor that locks SERCA in an E2-like conformation (Aguayo-Ortiz and Espinoza-Fonseca 2020). In the presence of TG, SERCA exhibited minimal sensitivity to changes in Ca^2+^ or nucleotide, indicating a collapse of the conformational ensemble into a single dominant population. These findings are in line with functional and crystallographic studies showing that TG stabilizes SERCA in a ‘dead-end’, low Ca^2+^ affinity intermediate, blocking Ca^2+^-dependent activation of the pump and inhibiting the structural transitions necessary for ATP hydrolysis and Ca^2+^ transport. While X-ray crystallography studies have suggested that TG populates a ‘closed’ headpiece conformation (Winther et al. 2010), the TCSPC data indicate TG stabilizes SERCA in an ‘open’ conformation. Our findings agree with previous spectroscopy studies showing that while TG-bound SERCA samples at least two major conformations, TG predominantly shifts the equilibrium towards an open, dynamic structure of the pump (Pallikkuth et al. 2013).

CPA is an inhibitor that binds near the opening of the gate between the Ca^2+^-binding sites and the cytosol. A comparative analysis of more than 80 crystal structures showed that the binding of CPA to SERCA populates a structural state that is similar to that populated by TG (Aguayo-Ortiz and Espinoza-Fonseca 2020). Interestingly, we found that CPA exerted concentration-dependent effects on SERCA that are different from those induced by TG. In contrast to TG, CPA attenuates, rather than abolishes, Ca^2+^- and ATP-dependent structural transitions, while progressively shifting the population away from the closed state of the pump. TCSPC spectroscopy revealed that CPA modulates the relative occupancy of closed and open headpiece conformations in the presence of nucleotide, indicating that CPA does not abolish Ca^2+^ or ATP-induced closure of the cytosolic headpiece. This partial preservation of responses to Ca^2+^ and ATP is mirrored by the effects of CPA on Ca^2+^ affinity. At low concentrations, CPA had minimal impact on K_Ca_ in the presence of AMP-PCP, whereas higher concentrations produced a significant reduction in apparent Ca^2+^ affinity. Previous studies have suggested that CPA hinders access of Ca^2+^ to the transport sites (Sagara et al. 1992). However, our results suggest that CPA may reduce apparent Ca^2+^ affinity through redistribution of nucleotide-stabilized conformational states rather than direct interference at the Ca^2+^ binding sites. Nevertheless, alternative mechanisms cannot be excluded. For example, CPA may partially restrict access of Ca^2+^ to the transport sites, alter the coupling between nucleotide binding and Ca^2+^ binding, or stabilize intermediate conformational states with lower apparent Ca^2+^ affinity. These findings support a model in which SERCA activity is regulated through shifts in conformational equilibria rather than stabilization of discrete states, in line with recent conceptual frameworks for allosteric regulation of the pump (Viskupicova and Espinoza-Fonseca 2025).

Importantly, our findings indicate CPA impairs the response of SERCA to AMP-PCP, an observation that correlates with the hypothesis that CPA perturbs ATP binding to SERCA (Moncoq et al. 2007). Our TCSPC measurements also revealed that CPA significantly blunts the structural response of SERCA to AMP-PCP, in agreement with previous biochemical studies showing that CPA disrupts ATP-dependent activation and coupling in SERCA (Moncoq et al. 2007). Together, these findings support a model in which CPA allosterically disrupts the ATP-mediated activation pathway of SERCA, a regulatory mechanism reminiscent of the modulation of SERCA activity by its regulator phospholamban (Cleary et al. 2024). This mechanism stands in sharp contrast to TG, which enforces a ‘dead-end’, inactive conformation that inhibits Ca^2+^- and ATP-dependent transitions altogether and produces a largely flat, nonresponsive profile even at low concentrations. The ability of CPA to selectively destabilize high-affinity Ca^2+^ binding while maintaining conformational plasticity highlights a previously underappreciated mode of SERCA inhibition, in which catalytic competence is diminished through modulation of structural populations rather than conformational arrest.

In conclusion, we used TCSPC spectroscopy to define the distinct allosteric mechanisms of SERCA inhibition by TG and CPA. Whereas TG abolishes Ca^2+^- and ATP-dependent structural transitions and traps SERCA in an inactive state, CPA attenuates these transitions in a concentration-dependent manner while preserving conformational responsiveness. The ATP-dependent effects of CPA on Ca^2+^ affinity support an allosteric mechanism that perturbs the activation pathway rather than directly blocking ligand binding. Together, these findings establish a dynamic framework for SERCA inhibition and highlight TCSPC as a powerful approach for resolving mechanism in conformationally complex membrane proteins.

## Data Availability

All data are available in the main text.
